# Adjusting the input ultrasound image data and the atherosclerotic plaque detection in the carotid artery by the FOTOM^NG^ system

**DOI:** 10.1080/13102818.2014.924271

**Published:** 2014-07-10

**Authors:** Lačezar Ličev, Jan Tomeček, Radim Farana

**Affiliations:** ^a^ Department of Computer Science, Faculty of Electrical Engineering and Computer Science, VŠB-Technical University of Ostrava, Ostrava-Poruba, Czech Republic; ^b^ Institute for Research and Applications of Fuzzy Modeling, University of Ostrava, Ostrava, Czech Republic

**Keywords:** active contour, anisotropic diffusion, atherosclerosis, Chan Vese, phase correlation, gradient vector flow, Hough transform

## Abstract

Stroke is the third most frequent cause of death. Specifically, ischemic stroke accounts for the largest group of this kind of cases. Despite all the advances in medical therapeutic methods, no methods that would reliably reduce mortality from ischemic stroke have been found. Prevention is still the most significant way to combat stroke. When the frequent cause of ischemic stroke is atherosclerotic plaque in the carotid artery, its exploration can help to determine the development of the disease. These problems were very extensively discussed in October 2013 during the XVI International Neurosonology Congress in Sofia organized under the auspices of World Research Neurosonology Group, Bulgarian Neurosonology and Cerebral Hemodynamics Association. Our goal was to develop special modules for carotid artery picture processing (AVI file processing, reparation and reconstruction) and modules containing tools for automated carotid artery plaque detection; and to solve its measurement and three-dimensional modelling of the carotid artery and the plaque. New modules were implemented into the FOTOM^NG^ system and tested on appropriate input data files, which verified their functionality and applicability.

## Introduction

Stroke is the third most frequent cause of death. Specifically, ischemic stroke accounts for the largest group of this kind of cases. Despite all the advances in medical therapeutic methods, there have still not been found methods that would reliably reduce mortality from ischemic stroke. Prevention is still the most significant way to combat stroke. When the frequent cause of ischemic stroke is atherosclerotic plaque in the carotid artery, its exploration can help to determine the development of disease. These problems were very extensively discussed in October 2013 during the XVI International Neurosonology Congress in Sofia organized under the auspices of World Research Neurosonology Group, Bulgarian Neurosonology and Cerebral Hemodynamics Association.[1]

Duplex ultrasonography is mostly used to examine the carotid artery thanks to its accessibility, non-invasiveness and possible repeatability. A disadvantage of this method is the limited length of the artery which can be investigated, and often subjective evaluation by the physician. Doppler methods are used to measure the blood flow through the artery and they can also help to determine the reduction of the lumen diameter. For the purposes of assessment of atherosclerotic changes, even if they do not prevent the stenosis, as well as the structure of sclerotic plaques, we still use the B-mode.

Our goal was to develop special modules for carotid artery picture processing (AVI file processing, reparation and reconstruction) and modules containing tools for automated carotid artery plaque detection; to solve its measurement and three-dimensional (3D) modelling of the carotid artery and the plaque. New modules were implemented into the FOTOM^NG^ system and tested on appropriate input data files, which verified their functionality and applicability.[2,3]

## Materials and methods

### Background

Due to the frequent occurrence of ischemic stroke, attempts have focused on early diagnosis. Detection at an early stage is a key factor in preventing fatalities. Despite the great advantages such as availability, speed, and non-invasiveness, which examinations using ultrasonic techniques provide, for many years there have been on-going efforts to improve the quality of these examinations, speed up the time to examinations or automate this activity. Most innovations in this area come from the fields of image processing and analysis.

Pre-processing and segmentation of longitudinal ultrasound images of artery are discussed in [4]. Segmentation with the ellipse placed by user is described in [5]. In [6] detection also based on Hough transform (HT) followed by gradient vector flow (GVF) based segmentation.[7] Segmentation by parametric active contours are also used in [8] Similar image pre-processing and morphological operations based segmentation is described in [9]. Measuring the degree of narrowing is engaged in [10]. Detection using a modified Viola–Jones algorithm were proposed in [11] and segmentation of artery by active shape model [12] was introduced in [13].

### Analysis of carotid artery video and electrocardiogram records

#### Electrocardiogram recording and analysis

Since the recording artery probe moves about 1 mm with each heartbeat, it is necessary to select the record of the ultrasound images which depict the state of the arteries in a particular recurring time-interval (i.e. the time between any two heartbeats), because expansion and shrinkage of the vessels occurs again at each interval. The objective is to capture the artery in the same extension as in the previous interval, although in practice the intervals are not of equal length. It is therefore necessary to design and create a module for automatic identification of such events in order to minimize possible human factor.

Electrocardiography (ECG) is a technique for heartbeat recording. Electrodes associated with a recording device (electrocardiograph) are placed on the skin of the four limbs and the chest wall. The recorded electrocardiogram is drawn as a heart activity record on a moving paper strip. It is usually used in the diagnosis of heart disease, which can cause typical ECG changes.

**Figure 1.**
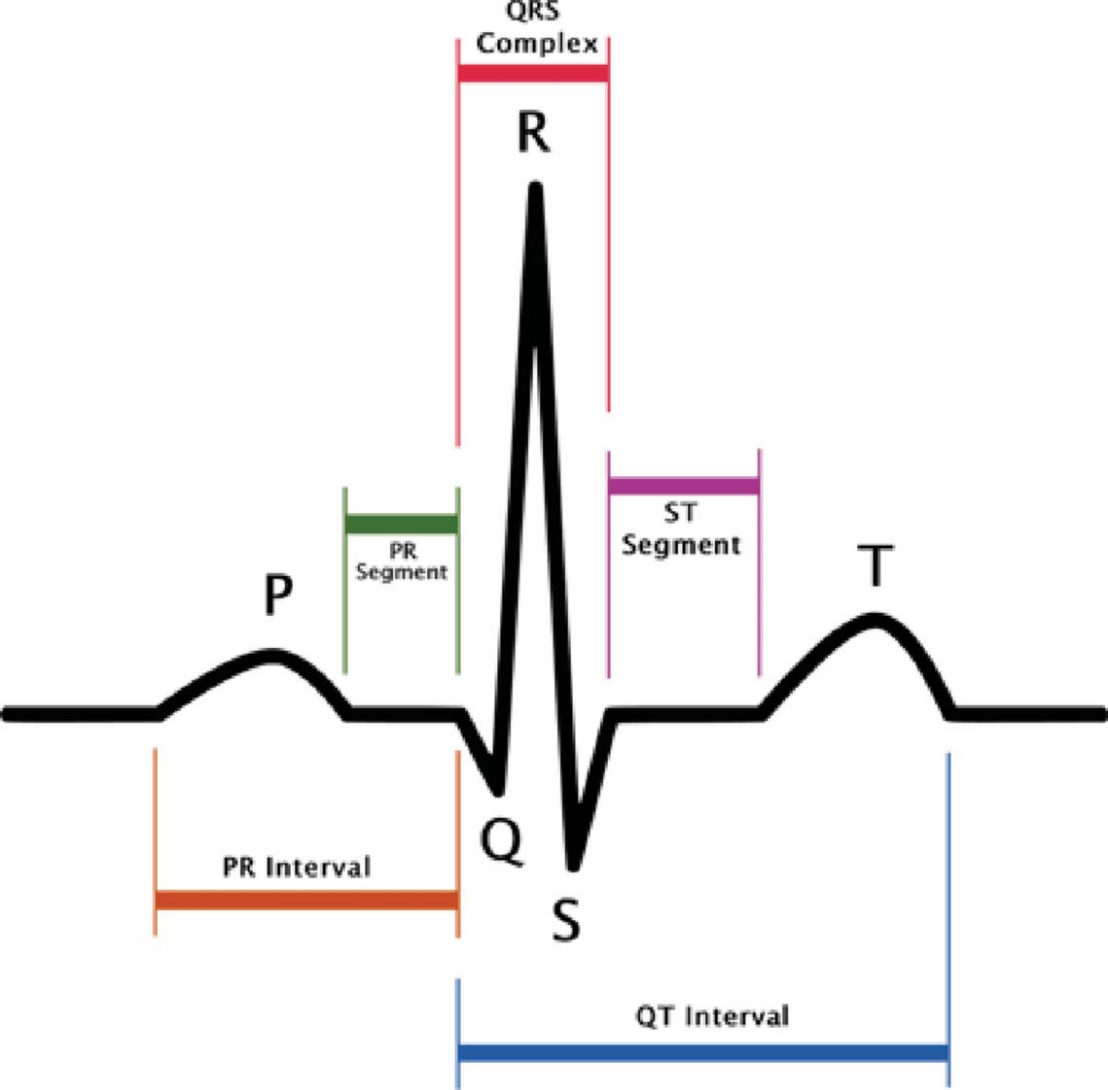
Normal ECG signal course.

The impulse for myocardial contraction arises in the sinoatrial (SA) node in the right ventricle, where it spreads further. For the purpose of our brief interpretation, it is important to note that this primary signal is so weak that it is not recorded during normal ECG. The first signal wave, which can be seen on the ECG (Figure 1), the P-wave, which testifies to repolarization of the ventricles, thus testifies the beginning of their contraction. The repolarization of the ventricles cannot be recognized on ECG as a relevant biosignal, because it is overshadowed by a much higher signal originating from ventricular depolarization.[14] This signal is characterized by a complex of QRS waves. The following T-wave indicates the next ventricular repolarization.[14]

Automatic recognition of the ECG graph area is very difficult and unreliable and therefore the user has an instrument which marks the area where the chart is located and only this area will be processed. It will be a classic rectangular area selection tool with which the user has certainly become acquainted with in many other programmes. Mainly the processing speed is the advantage of working with a smaller selected area.

**Figure 2.**
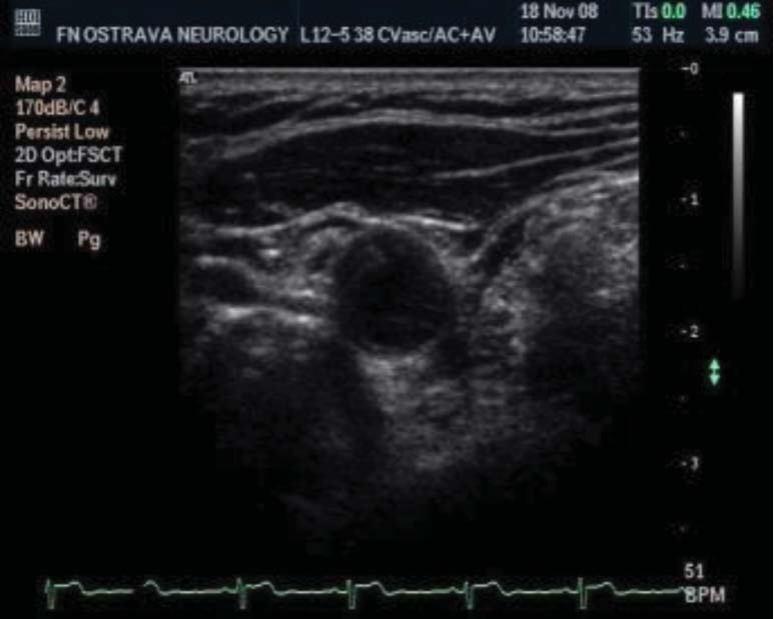
Ultrasonic picture taken from the video signal.

Therefore, the gap which redraws the graph is searched. In the first picture, always similar to Figure 2, it is therefore necessary to go through the whole composition and to search a rendered graph in each column. If this place is found, we move to the next column and the search is repeated. However, if the graph line is not found in the column, it means that there may be a redrawing gap, but it can also be a fault. That is why it is suitable if the image is pre-processed by thresholding before finding the procedure. Thresholding is a function that adjusts the input values generally by formula.[15]

**Figure 3.**
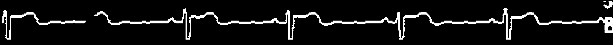
ECG graph pre-processed by thresholding.

The threshold value was adjusted to value 72 after long research. This pre-processing is responsible for eliminating the background noise and highlighting the graph (Figure 3). However, artificial holes and inconsistencies in the graph can arise, as a side effect.

**Figure 4.**
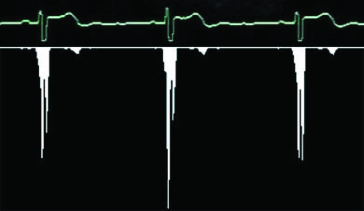
Variance computation.

When the redrawing hole is found, the algorithm remembers several columns to the left of the hole and goes in search of the next picture. Finally, all parts are connected together and a long strip with the corresponding graph is created. As mentioned before, our situation is highly complicated by the letter which covers the graph and therefore it is impossible to find the redrawing hole in this place, if being there. If the algorithm cannot find the redrawing hole in any space, it is considered that the hole is hidden behind that letter and therefore only an empty space is saved. As a result, the strip chart includes a few gaps, which can cause problems if oscillations of the QRS complex (see Figure 1) need to be drawn at this point. The following text shows how to search for single QRC complexes in the strip graph that correspond to the periods and how the heart rate is measured on the basis of these periods. We can use many different algorithms for QRS complex detection. All of them, however, presume an exact input signal but not a low-quality graph picture with low resolution. Then we develop a special algorithm for QRS complex detection in the low-quality picture. It is based on a calculation of the sample variance. It is obvious that for linear parts of the graph the dispersion will be very small; on the other hand, the dispersion will be much larger for the oscillating parts of the graph. The variance is determined for a small part of the graph, for example, a 5-pixel window, which is moved by 1 pixel right for the next computational cycle. We obtain a number which expresses the graph variance for any of these windows. Figure 4 shows the columnar graph of the computed variance values for a small part of the ECG graph. Now we have a set of variance values and we will find the first biggest among them. Its serial number represents the place of the QRS complex.

When all QRS complexes are found, we can easily separate the graph to individual periods. When a user determines that they are interested in a specific location on the graph, the algorithm recalculates this place relatively to the position at that individual period and this relative position is highlighted to all other periods. This is done to ensure that all individual periods will be of the same length. For example, the heart rate speeds up during intensified physical strain, resulting in a shortened period. Now the areas of interest are highlighted and the last step is to map them to the frames which could be saved as individual pictures for processing.

#### Input image data adjustment for objects detection improvement

The main goal of the new module for video or measurement image combination development is the subsequent processing and 2D correction of the object located in the picture. The method for picture combination used for high-quality image creation is described below.

The module development is divided to two logical parts. The first part deals with image analysis and correction to create high-quality images for later use. The second part analyses and makes correction of geometric objects in images by the FOTOM^NG^ system.

Testing was done on proofing images and video files. The average processing time of one 26-second video file comprising 671 images took 1.5 h on a single thread, which means that the processing time for two images lasts from eight to nine seconds. Determining the size of the offset between two images took from seven to nine seconds on average. Phase correction took 70% of the total processing time on average.

Correction quality and success rate depend primarily on appropriately selected reference frames. The difference in histogram intensity or contrast between the reference image and the defective image should be as small as possible in order to properly determine which missing part of the image might be completed by an average value or user-defined colour. Another important factor is the range of image deformation where the object of interest is deformed exceeding its own boundary in the correct condition, which can lead to completion of parts that do not require correction. This then leads to poor correction quality and correction can be described as a failure.

**Figure 5.**
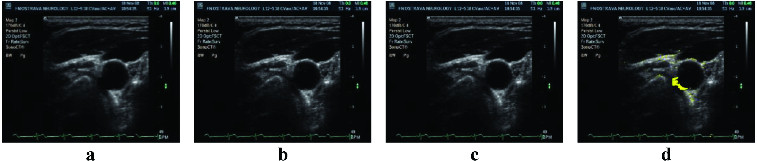
Image with missing artery parts (a); artery part repaired using a reference image and the image with missing parts artery for correction (b); repaired artery parts, completed by an average of the values of reference images (c); fixed artery part with a user defined colour (d).

**Figure 6.**
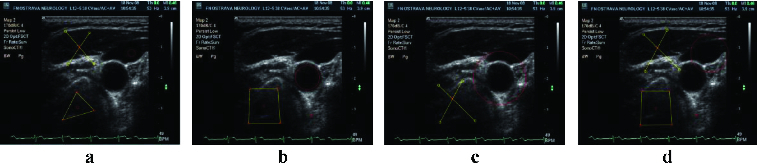
Image with objects designated for correction (a); reference image number 1 (b); reference image number 2 (c); repaired objects in the image when the objects of reference images 1 and 2 are used (d).

The second testing part was carried out on images containing FOTOM^NG^ objects with poorly defined characteristics or the image without desired objects. These objects were completed or adjusted to the reference image objects. Figures 5 and 6 show the first testing results of this module.

### Image analysis and carotid artery detection

#### Image pre-processing

**Figure 7.**
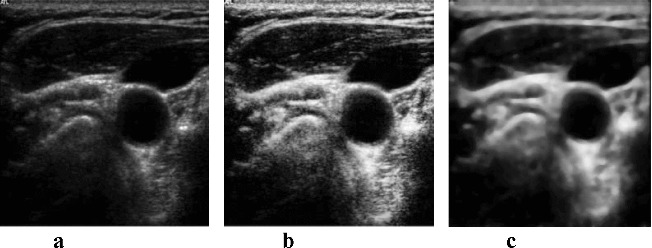
Original image (a); image after classical histogram equalization method (b); image after CLAHE application (c).

Although the dynamic range and brightness (consequently to all of incoming gains and TGC) are modified in the utrasonograph, the contrast and brightness of the analysed images are very low.[16–18] One of the first steps of image processing is the image contrast adjustment. Although the contrast adjustment is mainly used before visual image evaluation, image adjustment by contrast-limited adaptive histogram enhancing (CLAHE) [19] has led to overall better results in the final artery detection (Figure 7). The principle of this method is to limit the declination of the transfer function, thus the declination of the distribution function.

The next pre-processing step is the speckle noise filtering.[20,21] During testing, it was experimentally determined that for subsequent edge detection and HT, the conventional median filter seems to give the best results.[22]

Edge detection is done by a classical Sobel filter, followed by an iterative adaptive thresholding method.

The HT has been used for artery detection in the edge image, especially HT for circle detection. Although the artery does not always have a circular shape, results obtained by artery detection by HT for circle detection were much better in comparison with the generalized HT detection and also with less computational complexity.

#### Artery reconstruction

An acoustic shadow artefact appears in most of the analysed carotid artery ultrasonic images, especially when calcified plaque is present at the top of the artery. In this case, information on a large part of the artery side is missing, which results in unwanted contours development outside the area of interest. From the viewpoint of parametric active contours,[23] it is possible to prevent the loss of contour using greater model stiffness to a certain extent. Greater stiffness, however, causes deterioration of the contour properties in the concave areas models, particularly in the area of the plaque. When GVF is used as external energy of the edge, the artefacts raised from the acoustic shadow at the edges of the artery sides are a source of the forces that pull the contour outside of the area of interest. It is therefore necessary to reconstruct the missing part of the artery side. Two algorithms have been proposed for this purpose.

**Figure 8.**
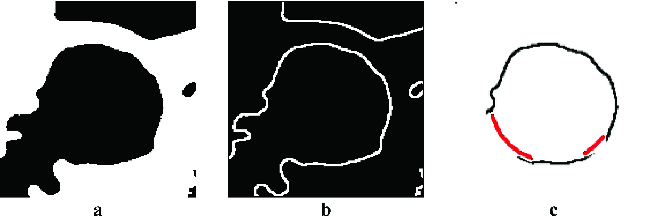
Smoothed image after cropping and threshold (a), after edge detection (b) and after artery side reconstruction (c).

The first principle uses ‘image stitching’. The second method for partial artery object reconstruction uses the actual image only. An algorithm which converts the edge image into polar coordinates is used when the centre of the circle found is used as the coordinate origin. The minimum distance for the given angle is taken into consideration. Two values are selected, one as a threshold for the maximum distance, when the artery side is not completed, and the second as a distance for adding pixels, when the carotid side is not found for this angle. The first value was experimentally determined as *t* = *r* + 4, where *r* is the radius of the circle found in pixels. The second value was determined equal to *r*. Figure 8 presents the result of this reconstruction method.

The next step is to prepare the image for parametric active contour segmentation. The image after the median filter application is cropped around the determined centre to maintain only the artery surrounding for further processing. Then an adaptive iterative threshold method is applied, in this time with a modified threshold boundary shifted by brightness value 10 towards higher intensity values. This value was also set up experimentally. Subsequently, the edges are detected.

#### Inner artery side segmentation

**Figure 9.**
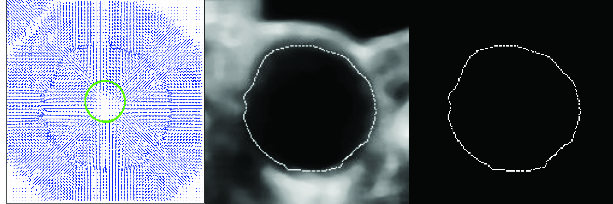
The resulting polygon after inner artery side segmentation.

Inner artery side segmentation (Figure 9) is achieved by a parametric active contour.[24,25] The result of the segmentation is a contour that defines the inner side of the artery. When determining the faultless artery outline, it is then possible to determine the content and size of the plaque by the difference between the determined outline of the artery and the contour.

#### Implementation and testing

As in the case of subjective evaluation of ultrasonic images, here the implemented algorithms for the carotid artery detection and analysis also primarily depend on the quality of the measured data.

The algorithm for automatic artery detection mainly depends on the surrounding structures in the image, which may be of a part-circular shape. In this case, if the artery object is not very evident, incorrect object detection often occurs. Another problem appearing using a mechanical measurement device is occasional compression of the artery by the probe and thus image deformation.

For the purposes of testing, 1100 images obtained from videos taken by the acquisition unit were used. The artery was correctly found in 870 images. The artery was identified incorrectly in 230 images.

**Figure 10.**
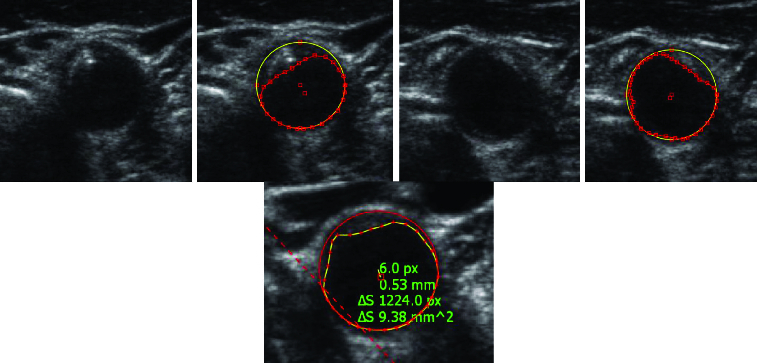
Sample result of the proposed algorithm for artery detection and segmentation.

Proper detection was significantly dependent on the time of testing; the detection success dropped down with time, and thus with the probe shifting. Given the character of the test images, it is obvious that this happened because the ultrasound probe is/becomes put out of tune. At the beginning of the measurement, the probe is positioned by a doctor so that the artery is most pronounced in the image. In these cases the detection success rises up to 100%. Even the presence of large calcified sclerotic plaques does not often lead to greater error detection. Over time, as the probe is moved automatically, the detection capability of the algorithm degrades with degrading visual quality of the object. See Figure 10 for examples of result.

## Results and discussion

The development of a video-processing module provides the ability to load and play videos of various formats. It becomes a powerful tool designed mainly to make the analysis of ultrasound records easier. The programme module provides basic functions of a video player and is able to choose and save individual images from a video file, based on the user settings, and analyse the video signal and identify their individual ECG periods, from the graph captured in the video recording. The user could also set up intervals if the periods were not detected or were detected incorrectly. When creating a video player, the emphasis was put on continual video replay even when an unexpected system load occurs.

The image and objects correction module provides an opportunity to correct objects in an image and to combine images to create a single high-quality image. It is possible to process images taken separately or from a video signal. Module functionality and usability was validated. Module functionality, accuracy and integration implication were confirmed during the testing process.

Segmentation results were compared with manually obtained artery sides. They were marked out directly by a doctor based on their training in image processing. Static ultrasound picture evaluation is very subjective in many cases. It is often impossible to differentiate between the plaque and the noise; the obtained result rather depends on the doctor's experience. Another complication is the fact that during a classical examination performed by a doctor, the parameters of the ultrasound probe could be changed, such as amplification (gain), frequency, etc. This is impossible when a static record is processed. Then we must work on limited information from the initial setup values. Despite these facts, segments considered as correct (reference) to measure the deviation of automatic segmentation were manually marked. The area that does not overlap with the reference regions was measured in square millimetres (mm^2^). Although it might not be only the plaque, these deviations were observed mainly because FOTOM^NG^ allows us to easily measure the area based on the difference between two object areas, i.e. the area of the contour of the artery side and inside the artery.

**Figure 11.**
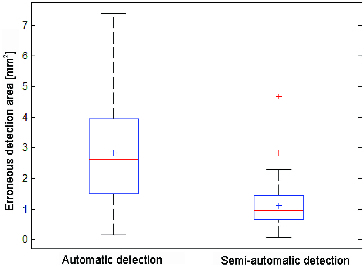
Detection error box plot.

It is evident that using the semi-automatic detection, when the user manually marks the area of the artery side, significantly more accurate results can be obtained. An average value of the defective marked area is 2.816 mm^2^ with variance of 2.593 mm^4^ for automatic detection, whereas for the semi-automatic detection the average value is 1.105 mm^2^ and the variance 0.585 mm^4^. Box plots with errors are shown in Figure 11.

## Conclusions

The algorithms applied for carotid artery detection and analysis primarily depend on the measured data quality. Although the image acquisition system brings many advantages for further processing, it lacks the ultrasonic parameter tuning which is done by a person during a standard measurement process, based on the current image quality and object resolution. Another problem is artery compression caused by the mechanical acquisition device, which causes image deformation. This situation significantly complicates automatic artery detection and correct artery side marking by the automatic detection algorithm. The algorithm for automatic artery detection mostly depends on the surrounding structures in the image, which may be of a part-circular shape. In this case, if the artery object is not very evident, incorrect object detection often occurs. We see the contribution of this work in more areas: first, in the development of the algorithm for automatic artery detection and segmentation and furthermore, in the process for the image object-reconstruction algorithm. It significantly improves the detection and segmentation results in the case of poor ultrasound image quality. Last but not least, the proposal of procedures for analysis of the AVI files taken by an ultrasound measurement probe was implemented in the FOTOM system. All of these tools have been implemented in the program FOTOM^NG^, which also allows quantitative evaluation of artery narrowing, area and volume of atherosclerotic plaque measuring, and 3D modelling. Together with the acquisition unit,[2,3] the FOTOM^NG^ system becomes a unique tool for the carotid artery examination. 
